# Olfactory Generalization in Detector Dogs

**DOI:** 10.3390/ani9090702

**Published:** 2019-09-19

**Authors:** Ariella Y. Moser, Lewis Bizo, Wendy Y. Brown

**Affiliations:** 1 Canine and Equine Research Group, School of Environmental and Rural Science, University of New England, Armidale, NSW 2351, Australia; wbrown@une.edu.au; 2 School of Psychology, University of New England, Armidale, NSW 2351, Australia; lbizo@une.edu.au

**Keywords:** canine, detection, discrimination, odor, scent, sniffer dog, target, training, variation

## Abstract

**Simple Summary:**

Dogs are valued for their odor detection capabilities in a vast range of fields. They help to find hidden and elusive targets, such as explosives, narcotics, missing persons, and invasive or endangered species, amongst an extensive list. In all these roles, dogs are required to find real target odors that vary somewhat from those with which they were trained. For example, dogs might be trained with an explosive mixture or certain explosive compounds, and then must be able to find homemade explosives of differing compositions or manufacturing processes. This ability, to respond to similar odors in the same way as they would respond to the originally trained odor, is known as generalization. A failure to generalize can result in dogs missing targets in working scenarios. Although generalization is usually desired to some extent, dogs must also discriminate against related odors that are not targets. Therefore, research that investigates factors that can influence dogs’ tendency to generalize, and conversely to discriminate, can inform training strategies to improve detection outcomes. However, this field requires further research with greater application to practical training.

**Abstract:**

Generalizing to target odor variations while retaining specificity against non-targets is crucial to the success of detector dogs under working conditions. As such, the importance of generalization should be considered in the formulation of effective training strategies. Research investigating olfactory generalization from pure singular compounds to more complex odor mixtures helps to elucidate animals’ olfactory generalization tendencies and inform ways to alter the generalization gradient by broadening or narrowing the range of stimuli to which dogs will respond. Olfactory generalization depends upon both intrinsic factors of the odors, such as concentration, as well as behavioral and cognitive factors related to training and previous experience. Based on the current research, some training factors may influence generalization. For example, using multiple target exemplars appears to be the most effective way to promote elemental processing and broaden the generalization gradient, whereas increasing the number of training instances with fewer exemplars can narrow the gradient, thereby increasing discrimination. Overall, this research area requires further attention and study to increase our understanding of olfactory generalization in dogs, particularly detector dogs, to improve training and detection outcomes.

## 1. Introduction

Olfaction may be the most fundamental sense for dogs, being optimized to perceive and comprehend the world around them in great detail. This remarkable olfactory sense is harnessed by humans to aid in the detection of an enormous range of elusive targets, including explosives (e.g., [1]), narcotics (e.g., [2]), missing persons (e.g., [3]), and invasive or endangered species (e.g., [4]), amongst an extensive list. Training dogs for these scent detection tasks usually entails training a behavioral response that is associated with a specific target odor (e.g., [5]). However, odors are typically comprised of a complex matrix of volatile organic compounds that vary somewhat, even between different examples of the same commodity. Since it is usually impossible to train with every potential variant of a target odor, detector dogs must learn to display the same behavioral response when they encounter novel variations of a learned odor.

To perform detection tasks, dogs must process a massive amount of olfactory information and respond to new and changing stimuli. An essential mechanism by which they do this is stimulus generalization, a phenomenon which allows organisms to categorize stimuli that are perceptually similar and thus likely to share associated outcomes [6,7,8]; which occurs in contrast to discrimination, allowing organisms to treat different stimuli as having differing outcomes [9]. Generalization and discrimination can, therefore, shape dogs’ perception of the odor stimuli that they encounter. In turn, this impacts their role as odor detectors by regulating how they respond to variations in target odors and discriminate these from non-target odors.

The tendency of dogs to generalize responding to odors other than one on which they were trained can considerably influence detector dog outcomes—too little generalization can lead to dogs missing targets, and too much generalization can lead to an increase in false responses. As an example, narcotics-detection dogs trained with pure cocaine samples must generalize their training by responding to cocaine variants containing different impurities, or of different origins or manufacturing processes. Conversely, they must be sufficiently discriminatory and refrain from responding to similar odors that pose no risk, such as snapdragon flowers, which emit the same primary odor compound as cocaine, methyl benzoate (C_8_H_8_O_2_) [10]. Depending on the desired detection outcomes for specific targets and the risks associated with either misses or false alarms, trainers might try to encourage dogs to be more general or more discriminatory. Olfactory generalization and discrimination are, therefore, critical factors in dogs successfully transferring learning from training contexts to authentic working contexts. However, currently, there is a shortage of research that explicitly investigates olfactory generalization in detector dogs.

## 2. The Generalization Gradient

**Figure 1:**
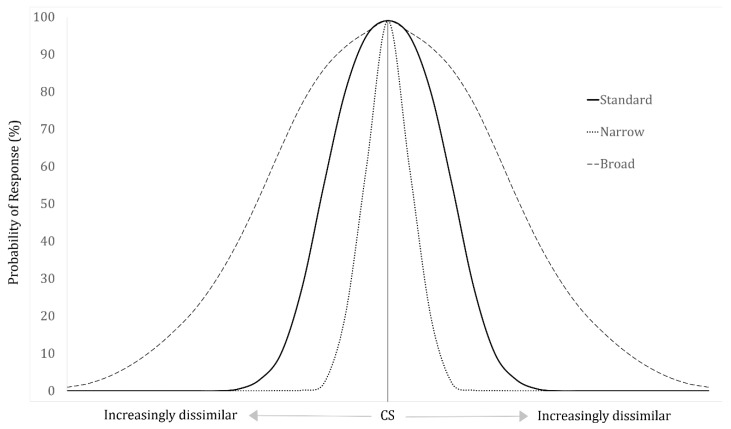
Three theoretical generalization gradients varying along one spectrum, illustrating the probability of responding to the conditioned stimulus (CS) at the peak, with graded decreases in responses as the stimuli become increasingly dissimilar. The narrow peak depicts the least generalization, with fewer responses to stimuli as they deviate from the CS. The broad peak depicts the most generalization, with the greatest probability of responding to stimuli differing from the CS.

Plotting response frequencies to similar stimuli after conditioning produces a “generalization gradient” [11]. This is usually depicted as a Gaussian curve with the response probability on the *y*-axis, with the peak centered around a conditioned stimulus, where animals have had the opportunity to respond to similar stimuli on the same dimension [8,12]. The curve shows an orderly decrease in the probability of responding as the stimuli become increasingly dissimilar to the conditioned stimulus (see Figure 1). Essentially, this gradient illustrates an animal’s sensitivity to variations of a learned stimulus. As such, the width of a generalization gradient reflects the extent to which an animal has generalized learning to other similar stimuli along a continuum; a broad category encompassing more difference indicates greater generalization, while a narrow and more specific category indicates greater discrimination [8]. This same curve is predictable even for complex stimuli that vary along more than one dimension when those dimensions are transformed into a single axis [13,14]. Generalization gradients are useful to conceptualize and predict generalization and discrimination of olfactory stimuli, including complex odors and mixtures. The slope of the gradient also conveys the ability of an animal to discriminate between stimuli—the steeper the slope, the greater the level of discrimination and the flatter the slope, the greater the level of generalization.

Generalization gradients have been observed relatively consistently across a range of species and stimulus dimensions of different sensory domains. Many of the first explicit observations of the generalization gradient involved pigeons responding to different wavelengths of light (e.g., [9,12]). Since then, generalization gradients have been demonstrated in many different perception tests with a range of species, from humans and vertebrates to invertebrates. For example, some stimulus dimensions that have produced generalization gradients have included: Sound frequency (e.g., [15]), degrees of rotation (e.g., [16]), spatial location (e.g., [17]), shapes (e.g., [18]), and monomolecular odors (e.g., [19]). Moreover, generalization gradients can be predictive of responses to multi-dimensional, complex stimuli, such as perceived aggressiveness [20] and trustworthiness [21] in humans. Similarly, this same pattern likely governs canine responses towards complex odor variations.

## 3. Generalization of Structurally-Similar Compounds

As most organic odors vary greatly and in many dimensions, it can be preferable to reduce test stimuli to one variable dimension to investigate generalization systematically along a continuum [22]. Consequently, many tests of olfactory generalization and discrimination use monomolecular odors—a homologous series of aliphatic compounds that differ in one structural dimension, such as the addition or subtraction of carbon atoms (e.g., [23,24,25,26]).

Animals tend to generalize between compounds with the same and similar carbon-chain lengths. Hall et al. [27] observed this in an experiment in which they trained dogs to detect 1-pentanol (C_5_H_12_O) and then tested whether the dogs could discriminate between 1-pentanol and other alcohol compounds that differed only in their carbon chain length. They observed that dogs had the most difficulty discriminating 1-pentanol from compounds with only one carbon difference, with better discrimination in a systematic pattern as carbon additions or subtractions increased. This suggests that dogs likely generalize, rather than discriminate, between more structurally-similar compounds. Similarly, a recent report found that dogs responded to test compounds with differing carbon chain lengths at a lower rate than the initially trained compound, but observed high rates of generalization between compounds of different functional groups, including a ketone, alcohol, aldehyde, and ester, with the same carbon chain length [28]. This generalized response is likely caused by structurally similar compounds activating overlapping olfactory glomeruli, which in turn engender a generalized behavioral response [29,30]. The same neural process could govern generalized responses for more complex organic odors. This systematic approach to olfactory generalization testing can, therefore, inform theories and investigate factors that alter the generalization gradient.

Research using homologous series of compounds have highlighted some factors which appear to regulate the olfactory generalization gradient in other species. For example, the generalization gradient tends to narrow with increased learning instances about a conditioned odor stimulus, as the animal forms a stronger association with a stimulus that has had a very predictable outcome, thereby increasing specificity [31,32]. This finding highlights a potential issue of excessively or exclusively training with one particular exemplar, particularly if generalization is desired.

**Figure 2:**
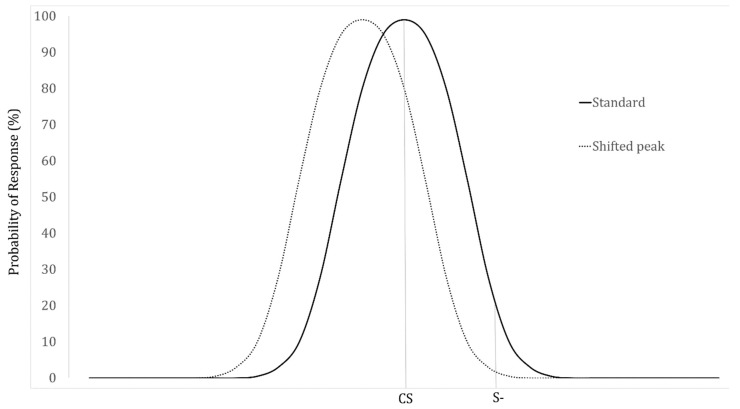
Theoretical peak shift of a generalization gradient. The gradient is shifted away from the negative stimulus (S-) so that fewer responses are made towards it. In the shifted peak, the highest rate of responding is no longer to the conditioned stimulus (CS).

Furthermore, discrimination training, wherein the animal is trained not to respond to a non-target stimulus, is known to create a “peak shift” that moves the peak in a direction biased away from the non-reinforced stimulus, which occurs in the generalization gradient of olfaction as well as other sensory domains (see Figure 2) [33,34,35]. This peak shift means that animals will respond to stimuli that are further from the non-target stimulus, even if that sometimes inhibits responses to the conditioned stimulus itself [36]; this is likely due to an overlap of the excitation and inhibition gradients [8,11]. Therefore, to help reduce peak shift and possible target misses, the early stages of discrimination training could potentially involve only odors that are very dissimilar to the target odor, making the contrast more clear [37]. Another approach that could counter this is “errorless discrimination training,” which was initially studied by Terrace [38] with pigeons, and found to eliminate peak shift. Gadbois and Reeve [39] have described this training method with dogs, in which the dogs are not specifically non-reinforced for an incorrect odor, but the odor is “faded in” incrementally, paired with the target odor so that they learn to ignore it.

## 4. Generalization of Mixtures and Components

Generalization becomes less straightforward when considering mixtures made up of two or more odor compounds. Olfactory research in several species, from spiny lobsters (e.g., [40]) and rats (e.g., [41]) to humans (e.g., [42]), has been carried out using simple mixtures of odorants to determine whether animals can generalize to their separate components and whether they can generalize from components to mixtures. This research can shed light on generalization to the shared components, or the “odor signature”, of complex target mixtures. Moreover, generalization between components and mixtures is directly relevant for dogs trained to detect explosives or other highly variable targets that are comprised of a multitude of individual odors, but for which they can often be trained using just one or a few major odor compounds that are present across all target variants (e.g., [1,43]).

**Figure 3:**
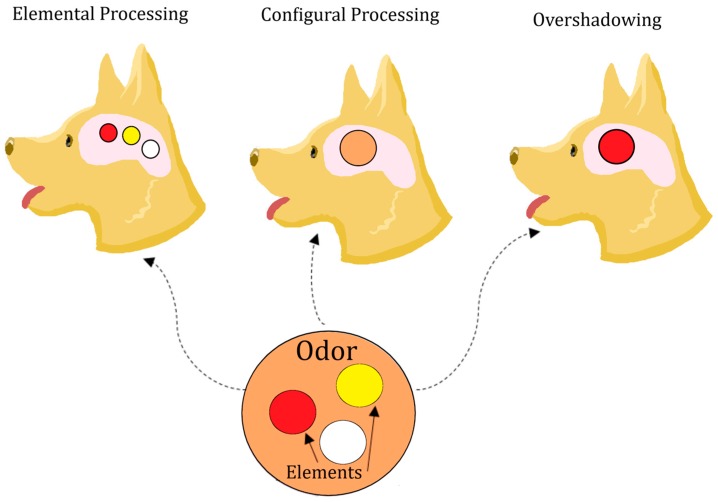
Illustration of some potential processing methods of an odor mixture. Elemental processing shows the dog perceiving all three elements or components; configural processing shows the dog perceiving the odor in its entirety but not its separate elements; overshadowing shows a type of elemental processing in which one salient component is perceived while the others are not. These different perceptions reflect what the dog would be likely to respond to after training with this odor stimulus.

Processing of odor mixtures often occurs in two different ways: Elementally or configurally [44]. Elemental processing refers to an organism perceiving each component of a mixture as separate and identifiable. These components may be singular compounds or submixtures. Conversely, configural processing occurs when an organism perceives a mixture as a unique odor, having a different quality than merely the sum of its components. Most animals, including dogs, do both under different circumstances [45,46,47]. Elemental processing can facilitate generalization between mixtures and components, whereas configural processing can prevent it [48] (See Figure 3).

Overall, despite it being a standard training practice, many dogs appear to have difficulty in generalizing to mixtures containing a particular component when trained on that component alone. For example, most dogs tend to struggle to generalize to explosive mixtures that contain ammonium nitrate (AN, NH_4_NO_3_) when trained on just AN alone, though with considerable differences between individual dogs [47,49,50]. Lazarowski et al. [49] found that dogs (n = 15) trained solely with AN showed, overall, only weak generalization to realistic AN-based targets, including fertilizer-grade AN (66.7% correct), AN combined with soil from the Middle East (73.3% correct), and AN combined with flaked aluminum (68.9% correct). Furthermore, there was high variability between individual dogs in their responses to mixtures. This trend was also observed when training solely with potassium chlorate (PC, KC_l_O_3_), after which only six of 16 dogs were able to generalize to PC-based mixtures containing accelerants and fuel to the satisfactory standard of 75% correct [51].

Dogs, like other animals, tend to generalize more readily to mixtures containing a common target component when they are trained with several different mixtures containing that component [52]. For example, Fischer-Tenhagen et al. [53] found that dogs (n = 2) trained with ten different herb mixtures containing chamomile, were able to generalize to novel herb mixtures containing chamomile better than those trained only with chamomile (n = 3), as measured by the proportion of correct responses (*p* < 0.001). Similarly, Hall and Wynne [47] studied the detection of two critical oxidizers in home-made explosives—AN and hydrogen peroxide (HP, H_2_O_2_)—one at a time in a cross-over design. They found that dogs (n = 4) were able to detect familiar and novel mixtures containing the target oxidizer better after training with several mixtures containing the oxidizer (post-training with just the oxidizer) than after training with just the oxidizer alone, as measured by the proportion of correct responses (*p* < 0.05). Both of these studies also found, however, that when training using several mixture exemplars, it took longer for the dogs to learn the task than using a single component, as it appeared to be a more difficult task [47]. Nevertheless, using several odor mixture exemplars in training may encourage dogs to process the mixtures elementally to define the specific shared odorant(s) that are associated with the reward.

On the other hand, encouraging dogs to respond to the separate elements of a target odor could unintentionally increase responses to benign odors with shared components or to odors that are systematically paired with the target (e.g., packaging or substrate). For example, explosive-detection dogs might generalize to common products that contain shared components, such as polyvinyl chloride (PVC) [54]. This potential over-generalization should be mitigated with explicit discrimination training against non-target elements that are common across training samples, such as packaging, substrates, or cutting agents; as well as against similar odor mixtures [55]. For example, in the case of explosive-detection dogs, training against non-target odor mixtures that are similar to explosive mixtures but do not contain an oxidizer might address this (e.g., [47]). This specific discrimination training would assist dogs in identifying the correct target element(s) and therefore prevent responses to non-targets.

A handful of studies have used dogs that were already trained to respond to certain target odors, and tested their responses to individual components of that target odor mixture, to identify which of those components may constitute the target’s “odor signature” (e.g., [2,54,56,57]). For example, researchers have found that dogs trained to detect cocaine will respond to just methyl benzoate, a decomposition byproduct of a cocaine component [58]. However, experiments which seek to identify an “odor signature” to use as a canine training aid should be used cautiously. Although a dog may respond to a component after being trained with an odor mixture, this does not necessarily translate to a dog responding to that odor mixture after being trained with just that component. The studies mentioned above [47,53] highlight some potential issues of training with single components. Therefore, training aids that are proposed with this method must still be tested to determine dogs’ ability to generalize from them to the actual target mixture, while also discriminating against other odors that may contain the same component(s) [54].

Configural processing, on the other hand, becomes more likely as the number of elements in the mixture increases [40]. In these cases, when more components are included in a training submixture, the likelihood of the subject generalizing their responses to complete mixtures increases [40]. For example, Lazarowski and Dorman [51] found that dogs improved their performance in generalizing to novel explosive mixtures when presented with several different odor components, not solely PC, that would be part of a target odor.

Finally, a major factor in determining how a mixture is processed appears to be the specific odorant components and the interactions between them [30,59]. For example, dogs trained with binary mixtures appear better able to generalize to the individual components if the mixture is comprised of molecularly dissimilar compounds, as opposed to more similar ones [28]. This may be due to structurally similar compounds competing for receptor sites or causing lateral inhibition, thereby masking a component and making elemental processing, and therefore generalization to its separate components, more difficult [26,52,60,61]. Furthermore, some compounds may be more salient, perhaps due to differences in concentration or vapor pressure, which can overshadow other components in the mixture [40]. As such, there may be certain odor mixtures that are intrinsically more difficult to identify elementally.

Moreover, individuals can differ in their perception of odors by attending to different features of an odor mixture, sometimes resulting in different behavioral responses [49,62,63]. Similarly, the amount of detection experience of the dogs may improve their ability to process odors elementally and generalize to components [28]. These factors may explain reported discrepancies between the performances of individual dogs in different experiments (e.g., [28,49,50]).

## 5. Generalization of Similar Complex Odors

For most fields in which scent detection dogs are deployed, the target odors that the dogs are trained to detect are complex odorant mixtures of which the specific components are not necessarily known. Different target odor variations likely have common compounds that dogs learn to respond to against a background of varying compositions. Another way to describe this is that a proportion of similarity between an initially trained complex mixture can elicit a response to a novel mixture [45]. In this way, it is analogous to generalizing between simple mixtures and components. However, this form of generalization is more difficult to study systematically, since exactly how similar the target odor variations are, or the features by which dogs generalize between them is usually not known. As such, most research in this area reveals generalization only incidentally. However, generalization between target odor variants is crucial for detection tasks, and further research in this area is warranted. The importance of this was highlighted, for example, in a study in which dogs trained with pure trinitrotoluene (TNT, C_7_H_5_N_3_O_6_) samples did not successfully generalize to actual TNT targets, which were of different origins and varied in overall composition [64]. Similarly, Elliker et al. [65] found that dogs that had been trained to respond to several samples of prostate cancer urine then failed to generalize and respond to prostate cancer biomarker(s) in novel urine samples.

However, there are many examples of dogs successfully generalizing to target odor variations and related odors with differing compositions. For example, Wright et al. [66] found that dogs could generalize to novel odors containing accelerants after being trained on several exemplars containing different types of accelerants, discriminating these from mixtures which did not contain an accelerant. Another example was observed in an experiment in which dogs successfully responded to estrous scent in urine after only training with estrous scent in vaginal fluid [67]. Furthermore, wildlife detector dogs must generalize to target odors in the field that can vary widely from training samples [68,69]. In one study, a dog was trained to generalize between otter feces variants, from different individuals, sources (captive/wild), ages, and diets, while discriminating against feces of other mammalian species [70]. This dog was able to generalize to novel otter samples after being trained with only two variations of feces. This number of necessary training exemplars would likely vary depending on the target odor and perhaps between individual dogs (e. g., [71]). Additionally, wildlife detector dogs are sometimes trained to discriminate against other related species [72], while others are encouraged to generalize to related species (e.g., [73]). It is possible that the processes used (i.e., elemental vs. configural) and the degree of generalization achieved, is determined in part by the training methods used. In this regard, it would be beneficial for dog trainers to have an understanding of these underlying processes to best increase generalization to target variations, while concurrently increasing discrimination against similar non-target odors [7,8,9].

Odor training aids that serve as a proxy for an actual sample of the target (e.g., pseudo-scents, simulates) are increasingly used for convenience in the training of odor detection dogs [74]. These rely on dogs generalizing appropriately from the training aid(s) to a live target. For practical and logistical reasons, it is not uncommon for wildlife- and pest-detector dogs to be trained using a training aid, such as some material previously exposed to the target animal, which serves as a proxy for an actual specimen. Dogs’ reported success in finding specimens in the field after training with odor proxies is evidence of successful generalization (e.g., [75,76,77]). On the other hand, synthetic training aids, such as pseudo scents, have been questioned and criticized (e.g., [74,78]). One experiment found that a cadaver-detection dog, which had been trained with human remains, did not respond to commercially-available cadaver pseudo-scents, suggesting the dog did not generalize between them [74]. Theoretically, this may be because the dog trained with genuine human tissue attended to different components of the odor and therefore did not recognize that particular odorant mixture, or the training aid odor had a different overall quality to the cadaver odor mixture. Such discrepancies in the odors of training aids compared to real targets can impact the ability of dogs to generalize to genuine targets in the field.

To create more realistic odor training aids, some new methods have been proposed that aim to capture a more complex target odor submixture than commercial pseudo scents provide, to improve generalization to genuine targets. For example, Pfiester, Koehler, and Pereira [79] have suggested using a solvent extract of bed bug scent to improve bed bug detection. Additionally, Simon, Mills, and Furton [80] have proposed using fractions of volatile organic compounds from specimens by collecting them from a gas chromatograph. Both these studies observed that dogs that had been trained with the actual target specimens would respond to these proposed training aids; however, the effectiveness of training with them and testing dogs on actual targets has not yet been investigated.

Importantly, conditioning with multiple stimuli exemplars appears to broaden the generalization gradient [8], resulting in responses to more target odor variations. Researchers have found, for example, that when training rats to respond to cigarettes, they will respond to more novel brands and variations if they are trained using more exemplars [81]. Therefore, using multiple training aids and training samples is usually recommended to achieve a generalized response across target variations [82].

## 6. Conclusions

Understanding the generalization and discrimination tendencies of dogs to trained olfactory stimuli affords better awareness of how to alter their response patterns through training. This is valuable to detector dog operations, in which we must train dogs to respond to varying targets with both sensitivity and specificity. However, there is currently little research that explicitly and systematically tests olfactory generalization in detector dogs, meaning there are still gaps in the literature. Specifically, there is a need for further research that translates readily to practical application with working detector dogs to improve training and detection outcomes.

Current research suggests that dogs perceive odor differences in compounds differing in carbon-chain length in a graded fashion. This provides an opportunity for the systematic study of factors that might alter their olfactory generalization gradients, especially those factors that could be used readily in training and have been found to affect generalization in other sensory domains, such as motivation [83], intervals between training [84], and schedules of reinforcement [85,86]. Furthermore, there is evidence that elemental or configural perception can be dependent on the specific odorants in a mixture. Therefore, a greater range of odorants that are operationally-valid should be tested with dogs to determine whether they can generalize between them to actual target mixtures, particularly to create effective training aids such as pseudo scents. Finally, explicit testing of generalization in a range of relevant complex odor targets could be carried out, with a systematic approach to the analysis of errors. This might be combined with gas chromatography-mass spectrometry to determine degrees of odor similarity and compound identities to determine how dogs might generalize or discriminate between them.
